# Editorial: Ocular ultrasonography and optical coherence tomography in the optic nerve disease

**DOI:** 10.3389/fmed.2023.1161123

**Published:** 2023-02-28

**Authors:** Nicola Rosa, Gilda Cennamo, Maddalena De Bernardo

**Affiliations:** ^1^Eye Unit, Department of Medicine, Surgery and Dentistry, Scuola Medica Salernitana, University of Salerno, Salerno, Italy; ^2^Department of Neuroscience, Reproductive Science and Odontostomatology, University of Naples Federico II, Naples, Italy

**Keywords:** OCT, optic nerve, ultrasound, papilledema, optic disc drusen, intracranial hypertension

Several disorders can affect the optic nerve and their differential diagnosis can be challenging, requiring expensive or uncomfortable tests.

Most of them are characterized by optic disc discoloration or oedema.

To differentiate optic disc oedema, particularly when the bulge is mild or unilateral, can be challenging (1).

Nowadays the presence of OCT and ultrasound can represent useful tools to help the clinician in the differential diagnosis of this kind of abnormalities.

Among the causes of optic disc oedema, there is the intracranial hypertension (2).

Elucidation of its underlying disease and follow up may require expensive or uncomfortable tests or even invasive procedures like lumbar puncture.

In particular, ultrasound has been widely used for this purpose, but it requires knowledge and skill to give reliable results (3–5).

In this collection, the clinicians can find several articles that will help them to utilize ultrasound and OCT in the management of these cases.

In particular, the three review articles on this topic can provide a useful tool to understand the role of ultrasound in the diagnosis and follow up of papilledema (Vitiello, De Bernardo et al.; De Bernardo et al.; Vitiello, Salerno et al.).

In the article by Johnson et al., the performance of three ultrasound units, two pocket ultrasounds and one standard-sized portable ultrasound, will give information on their precision and accuracy (Johnson et al.).

In the article by Wang et al., the usefulness of a multimodal imaging in the differential diagnosis of a disease, which could resemble an optic disc elevation, such as a morning glory disc anomaly (MGD) in a 7-year-old boy is described (Wang et al.).

Optic tract lesions (OTL), another challenging topic usually caused by a tumor or aneurysm, less frequently due to trauma, inflammatory or demyelinating disease, are discussed in the article by Cohen-Sinai et al.. The authors present an algorithm to simplify the diagnosis of OTL based on findings derived by optic disc color photographs, visual fields, OCT scans, and MRI. This approach could simplify and improve the clinician's diagnostic capabilities in cases of suspected OTL (Cohen-Sinai et al.).

Optical coherence tomography (OCT), including the measurements of circumpapillary retinal nerve fiber layer (cpRNFL) thickness and macular ganglion cell complex (GCC) (RNFL + ganglion cell layer [GCL] + inner plexiform layer [IPL]), is the primary diagnostic method for glaucoma. The study by Nakakura et al. investigates the effect of epiretinal membrane (ERM) and a new associated parameter, the space between the ERM and retinal surface, on ganglion cell complex thickness in eyes with glaucoma, based on a matched comparison of visual field defects (Nakakura et al.).

Optical coherence tomography angiography (OCTA) is a promising new non-invasive ophthalmic imaging technology for visualizing and quantifying both peripapillary and parafoveal microvasculature, in particular it can visualize retinal vessels with the detection of motion contrast from the blood flow through the Superficial Capillary Plexus (SCP). In the article by Pugazhendhi et al. the authors, examining the peripapillary vascular structure in patients with non-arteritic anterior ischemic optic neuropathy, highlight how several vascular parameters could be good predictors of optic nerve and retinal changes (Pugazhendhi et al.).

Overall, the eight articles in this Research Topic: Ocular Ultrasonography and Optical Coherence Tomography in the Optic Nerve Disease, provide a broad discussion of recent insights into optic nerve diseases that covers the cause of altered assoplasmic flow as well as the mechanism of vascular damage.

We believe the collections are informative for basic researchers, clinical scientists, and patients who are affected by optic nerve diseases.

## Author contributions

NR and MD compiled/wrote the first draft. GC contributed to the outline. All authors reviewed and edited for final revisions and approved the final version for publication.
